# Acute Effect of Metformin on Postprandial Hypertriglyceridemia through Delayed Gastric Emptying

**DOI:** 10.3390/ijms18061282

**Published:** 2017-06-16

**Authors:** Daisuke Sato, Katsutaro Morino, Fumiyuki Nakagawa, Koichiro Murata, Osamu Sekine, Fumiaki Beppu, Naohiro Gotoh, Satoshi Ugi, Hiroshi Maegawa

**Affiliations:** 1 Division of Endocrinology and Metabolism, Department of Medicine, Shiga University of Medical Science, Seta-Otsu 520-2192, Shiga, Japan; dkst0310@belle.shiga-med.ac.jp (D.S.); fumiyuki-nakagawa@cmicgroup.com (F.N.); kmurata@belle.shiga-med.ac.jp (K.M.); sekine@belle.shiga-med.ac.jp (O.S.); sugi@belle.shiga-med.ac.jp (S.U.); maegawa@belle.shiga-med.ac.jp (H.M.); 2 Nishiwaki Laboratory, Cimic Biopharma Corporation, Nishiwaki 677-0032, Hyogo, Japan; 3 Department of Food Science and Technology, Tokyo University of Marine Science and Technology, Minato-ku 108-8477, Tokyo, Japan; fbeppu0@kaiyodai.ac.jp (F.B.); ngotoh@kaiyodai.ac.jp (N.G.)

**Keywords:** Metformin, postprandial hypertriglyceridemia, gastric emptying

## Abstract

Postprandial hypertriglyceridemia is a potential target for cardiovascular disease prevention in patients with diabetic dyslipidemia. Metformin has been reported to reduce plasma triglyceride concentrations in the postprandial states. However, little is known about the mechanisms underlying the triglyceride-lowering effect of metformin. Here, we examined the effects of metformin on lipid metabolism after olive oil-loading in 129S mice fed a high fat diet for three weeks. Metformin administration (250 mg/kg) for one week decreased postprandial plasma triglycerides. Pre-administration (250 mg/kg) of metformin resulted in a stronger triglyceride-lowering effect (approximately 45% lower area under the curve) than post-administration. A single administration (250 mg/kg) of metformin lowered plasma postprandial triglycerides comparably to administration for one week, suggesting an acute effect of metformin on postprandial hypertriglyceridemia. To explore whole body lipid metabolism after fat-loading, stomach size, fat absorption in the intestine, and fat oxidation (^13^C/^12^C ratio in expired CO_2_ after administration of glyceryl-1-^13^C tripalmitate) were measured with and without metformin (250 mg/kg) pre-treatment. In metformin-treated mice, larger stomach size, lower fat oxidation, and no change in lipid absorption were observed. In conclusion, metformin administration before fat loading reduced postprandial hypertriglyceridemia, most likely by delaying gastric emptying.

## 1. Introduction

Type 2 diabetes mellitus (T2DM) is a global epidemic [1]. Most individuals with T2DM have insulin resistance and are at increased risk of developing cardiovascular disease (CVD) [2]. Diabetic dyslipidemia increases CVD risk [3], especially in subjects with postprandial hypertriglyceridemia [4]. Small chylomicron remnants, generated from chylomicrons in the postprandial state, are recognized as a risk factor for coronary artery disease [5,6]. Therefore, postprandial dyslipidemia may be a good target for CVD prevention.

Metformin is the first-line therapy for the pharmacologic treatment of T2DM globally. Dyslipidemia in the insulin resistant state is characterized by elevated triglyceride (TG) levels because of increased hepatic very low-density lipoprotein production and impaired clearance of very low-density lipoproteins and intestinally-derived chylomicrons by lipoprotein lipase-mediated lipolysis [7]. In addition to conventional lipid-lowering therapies, such as fibrates, fish oils and statins, metformin, and pioglitazone are also effective for diabetic dyslipidemia [8,9,10,11,12]. It has been reported that combination therapy of fenofibrate with metformin reduced TG to a greater extent than fenofibrate monotherapy [13]. In contrast, pioglitazone had no additional TG-lowering effect when combined with fenofibrate [14]. These reports suggest that metformin may reduce plasma TG by mechanisms independent of improved insulin sensitivity. However, little is known regarding the fate of dietary fat following metformin treatment in vivo. In this study, we explored the mechanisms underlying the metformin-induced TG-lowering effect, particularly in the postprandial state.

## 2. Results

### 2.1. Metformin Treatment for One Week Decreased Postprandial Plasma Glucose Levels without Loss of Body Weight

**Figure 1:**
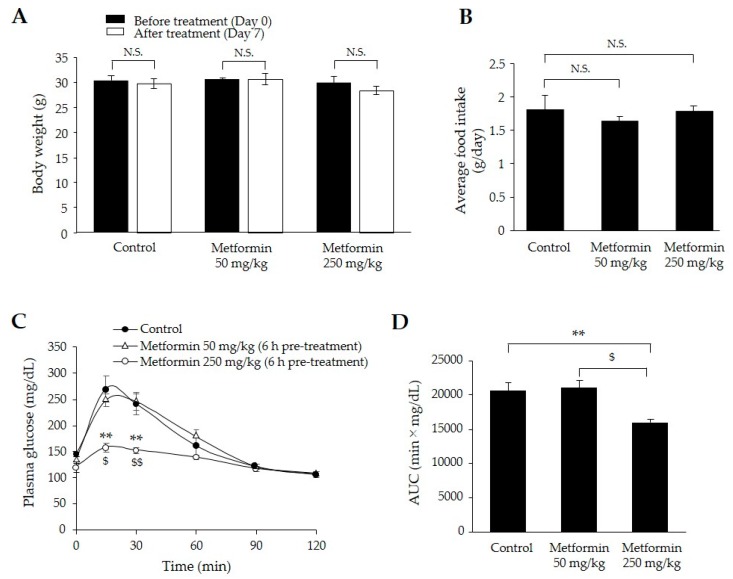

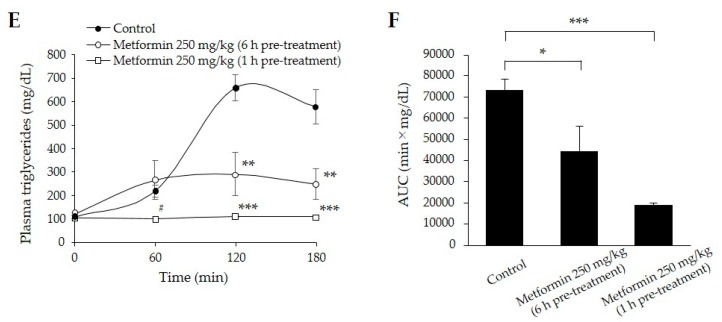
Effects of oral metformin treatment for one week in 129S mice. (**A**): Body weight change one week after metformin treatment (50 and 250 mg/kg); (**B**) average daily food intake; (**C**) plasma glucose concentration profiles 0–2 h after oral glucose (1.0 g/kg) administration. Metformin (250 mg/kg) was administered 1 h before glucose-loading; (**D**) area under the curve (AUC) for plasma glucose concentrations during the oral glucose tolerance test. Filled circle: control group (*n* = 5); open triangle: metformin (50 mg/kg) treatment group (*n* = 3); ope*n* circle: metformin (250 mg/kg) treatment group (*n* = 6) (**A**–**D**); (**E**) plasma triglyceride concentration profiles 0–3 h after oral olive oil (0.4 mL) administration. Filled circle: control group (*n* = 12); open circle: metformin (250 mg/kg) treatment 6 h before oral lipid tolerance test (OLTT) group (*n* = 6); open square: metformin (250 mg/kg) treatment 1 h before oral lipid tolerance test (OLTT) group (*n* = 7); (**F**): AUC for plasma triglyceride concentrations during the OLTT. Data represent means ± SE. * *p* < 0.05, ** *p* < 0.01, and *** *p* < 0.001 versus the control group. ^$^ *p* < 0.05 and ^$$^ *p* < 0.01 versus the metformin (50 mg/kg) treatment group. ^#^ *p* < 0.05 versus the metformin (250 mg/kg) treatment 6 h before OLTT group. N.S. indicates not statistically significant.

To determine the effective doses of metformin on glucose-lowering effect, we administered two different doses (50 and 250 mg/kg) for one week in mice fed a high-fat diet (HFD) for three weeks. There were no significant differences in body weight or food intake among the control, 50 mg/kg metformin treatment and 250 mg/kg metformin treatment groups (Figure 1A,B). After administration of two different doses of metformin for one week, we performed 1 g/kg oral glucose tolerance tests (OGTTs) in HFD-fed 129S mice. The 250 mg/kg metformin group displayed significantly lower plasma glucose concentrations at 15 and 30 min after glucose-loading than those in the control and 50 mg/kg metformin groups (*p* < 0.01) (Figure 1C). The mean OGTT area under the curve (AUC) in the 250 mg/kg metformin group was approximately 20% lower than those in the other groups (*p* < 0.01) (Figure 1D). Based on these results, we selected the 250 mg/kg metformin dose for further evaluation of lipid metabolism.

### 2.2. Metformin Treatment for One Week Decreased Postprandial Plasma TG

Pre-treatment with 250 mg/kg of metformin 6 h before the oral lipid tolerance tests (OLTTs) resulted in approximately 60% lower plasma triglyceride concentrations at 120 and 180 min compared with those in the control group (*p* < 0.01) (Figure 1E). Surprisingly, pre-treatment with 250 mg/kg of metformin 1 h before the OLTTs resulted in no increase in plasma TG levels during the OLTTs and the lowest AUC values among the three groups (*p* < 0.001) (Figure 1F).

### 2.3. Both Single and Long-Term Metformin Treatments Decreased Postprandial Plasma TG Levels

**Figure 2:**
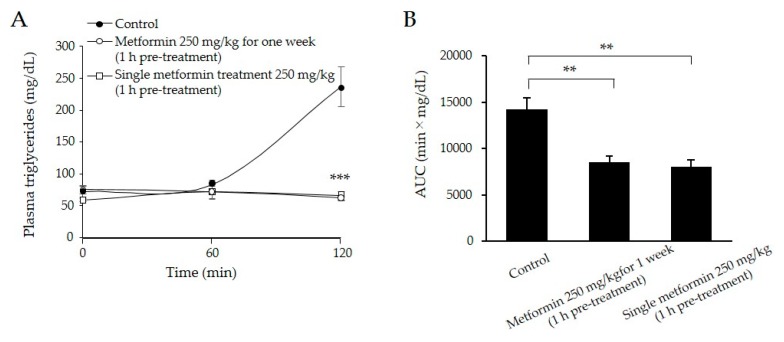

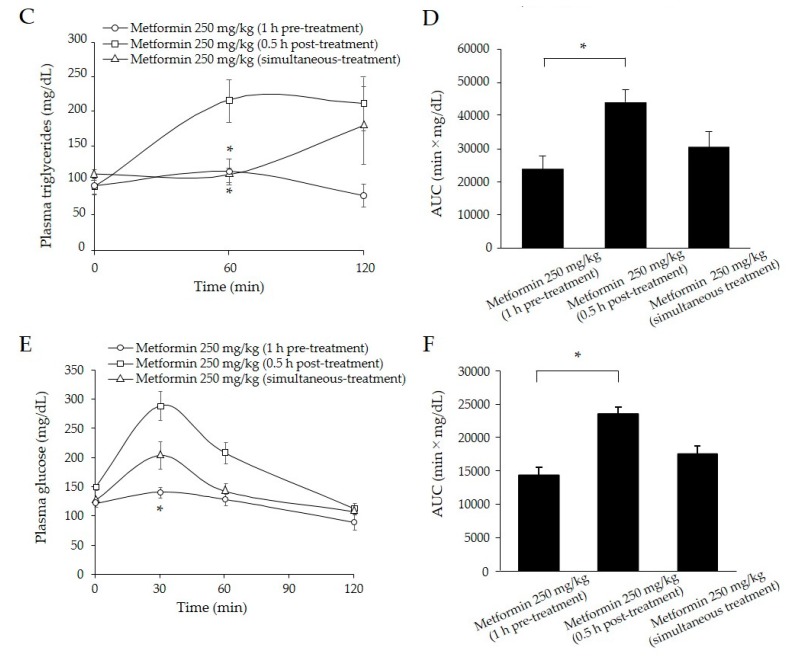
Effects of oral metformin treatment for one week (**A**,**B**) and a single metformin treatment (**C**–**F**) in 129S mice; (**A**) plasma triglyceride concentration profiles 0–2 h after oral olive oil (0.4 mL) administration. Metformin (250 mg/kg) was administered 1 h before olive oil-loading. Filled circle: control group; open circle: long-term metformin treatment for one week group; open square: single metformin treatment group; (**B**) area under the curve (AUC) for plasma triglyceride concentrations during the OLTT. Data represent means ± SE. *n* = 4 mice/group; ** *p* < 0.01, *** *p* < 0.001 versus the control group; (**C**) plasma triglyceride concentration profiles 0–2 h after oral olive oil (0.4 mL) administration. Open circle: metformin 1 h pre-treatment group; open square: metformin 0.5 h post-treatment group; open triangle: metformin simultaneous-treatment group; (**D**) AUC for plasma triglyceride concentrations during the OLTT. (**E**) Plasma glucose concentration profiles 0–2 h after oral glucose (1 g/kg) administration. Open circle: metformin 1 h pre-treatment group; open square: metformin 0.5 h post-treatment group; open triangle: metformin simultaneous-treatment group; (**F**) AUC for plasma glucose concentrations during oral glucose tolerance tests (OGTTs). Data represent means ± SE. *n* = 4 mice/group; * *p* < 0.05 versus the metformin (250 mg/kg) post-treatment group.

To examine the acute effect of metformin on lipid metabolism, we compared plasma TG concentrations between a single treatment and treatment for 1 week. Compared with the control group, both the single and one-week metformin groups displayed approximately 70% lower plasma TG concentrations 120 min after olive oil-loading (*p* < 0.001) (Figure 2A,B).

### 2.4. A Single Metformin Pre-Treatment Decreased Postprandial Plasma TG Levels Compared with Post-Treatment

To assess efficacy of the timing of metformin administration for postprandial TG levels, metformin (250 mg/kg) was administered 1 h before and 0.5 h after olive oil-loading. In contrast to pre-treatment, post-treatment of metformin displayed a two-fold increase in plasma TG concentrations 60 min after olive oil-loading (Figure 2C). The simultaneous treatment of metformin resulted in similar suppression of plasma TG concentrations 60 min after olive-oil loading, but a gradual increase at 120 min (Figure 2C). AUCs in the pre-treatment group were approximately 45% lower than those in the post-treatment group (*p* < 0.05) (Figure 2D). In OGTT, single metformin pre-treatment also significantly lowered plasma glucose concentrations than post-treatment (*p* < 0.05) (Figure 2E). AUCs in the pre-treatment group were approximately 40% lower than those in the post-treatment groups (Figure 2F). The simultaneous treatment of metformin showed intermediate levels of plasma glucose between post- and pre-treatments (Figure 2E,F).

### 2.5. Effect of Metformin on Gastric Size during Olive Oil-Loading

**Figure 3:**
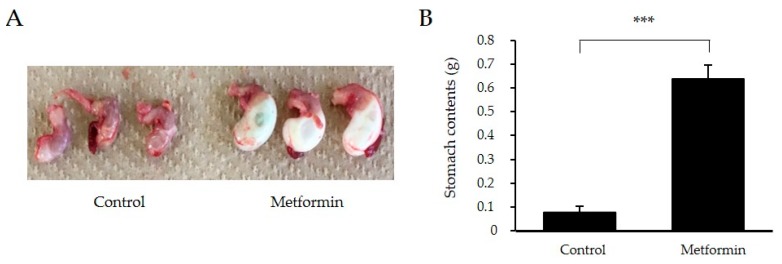
Stomach size and stomach content weights after olive oil administration in 129S mice following metformin (250 mg/kg) treatment (**A**,**B**). Data represent means ± SE. *n* = 4/group; *** *p* < 0.001.

To explore the mechanisms underlying the TG-lowering effect of metformin, 129S mice were sacrificed 3 h after olive oil-loading. Stomach sizes were larger in the 250 mg/kg metformin pre-treatment group than those in the control group (Figure 3A). Stomach contents were approximately seven-fold larger in the metformin pre-treatment group, suggesting that gastric emptying was delayed by metformin treatment (*p* < 0.001) (Figure 3B).

### 2.6. Metformin Did Not Affect Intestinal Lipid Absorption

**Figure 4:**
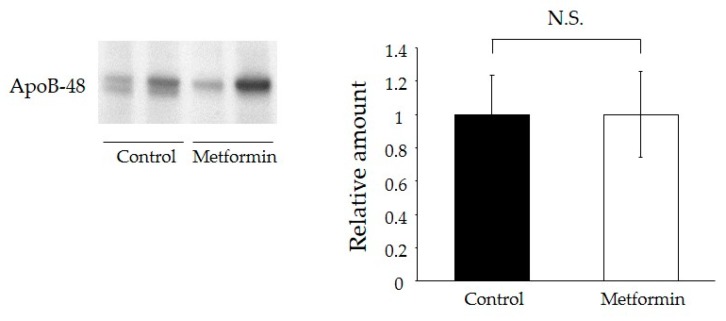
ApoB-48 secretion from isolated enterocytes of metformin-treated 129S mice into culture medium. Data represent means ± SE. *n* = 6–7 mice/group; N.S. indicates not statistically significant.

To evaluate the effect of metformin on lipid absorption, we measured secretion levels of apoB-48, a marker of newly synthesized chylomicrons, from primary cultured enterocytes. There were no significant differences in apoB-48 secretion between the control group and the metformin pre-treatment group (Figure 4).

### 2.7. The Metformin Treatment Group Had Lower Rates of Whole Body Fatty Acid Oxidation

**Figure 5:**
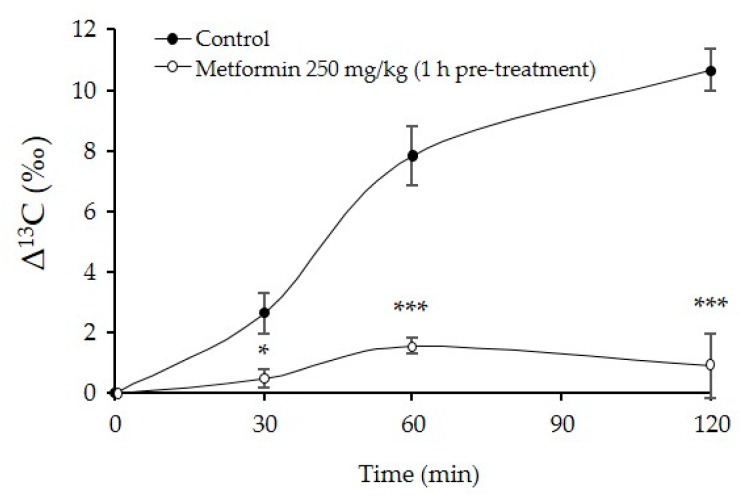
Changes in the ^13^C/^12^C ratio in expired CO_2_ after administration of glyceryl-1-^13^C tripalmitate in 129S mice following metformin (250 mg/kg) treatment. Data represent means ± SE. *n* = 5/group; * *p* < 0.05, and *** *p* < 0.001.

We evaluated the fate of lipids following metformin treatment using stable isotope labeled fatty acids. Glyceryl-1-^13^C tripalmitate was administered to 129S mice as a form of TG with or without 1-h metformin pre-treatment. Administered glyceryl-1-^13^C tripalmitate are beta oxidized and ultimately catabolized to ^13^CO_2_ in the mitochondria. Thus, changes in the ^13^C/^12^C ratio (Δ^13^C) in expired CO_2_ from mice indicated the degree of whole body beta oxidation. Metformin-treated mice displayed lower Δ^13^C increments than those of control mice (mean AUC Δ^13^C ‰, Control, 753.1 ± 61.9 (min*‰) versus Metformin, 112.6 ± 42.1 (min*‰), *p* < 0.001) (Figure 5). This result suggests that metformin treatment may delay gastric emptying and subsequently reduce glyceryl-1-^13^C tripalmitate absorption per unit time.

## 3. Discussion

In this study, we identified two novel outcomes of metformin treatment. First, a single pre-treatment with metformin reduced postprandial hypertriglyceridemia. Second, the mechanism underlying the TG-lowering effect may be mediated by delayed gastric emptying.

Metformin reduced postprandial hypertriglyceridemia in this study. This is consistent with previous clinical studies that have shown that metformin reduced plasma TG concentrations in the fasting and postprandial states [8,9,15,16,17,18]. In these studies, metformin treatment for several months increased insulin sensitivity because of improved long-term glycemic control and reduced body weight. Improvements in insulin resistance and reductions in body weight increase lipoprotein lipase activity [19,20,21], which plays a key role in breaking down plasma TG from TG-rich lipoproteins, such as chylomicrons and very low density lipoproteins. Therefore, it is difficult to distinguish the direct effect from secondary effects due to insulin sensitivity or body weight reduction. The TG-lowering effect, similar to the glucose-lowering effect, in the fasting state was reported to be dose-dependent [22]. In our animal studies, metformin treatment for one week reduced postprandial TG concentrations with no change in food consumption or body weight. Surprisingly, we found that a single metformin treatment had the same TG-lowering effect as metformin treatment for one week.

The mechanism underlying the TG-lowering effect may be through delayed gastric emptying. Medication efficacy can sometimes vary with the timing of administration relative to food intake. Meals decreased the bioavailability of metformin, with a 20% lower AUC and 35% lower *C*_max_, and delayed the absorption of metformin, with *t*_max_ prolonged by nearly 40 min [23]. However, post-meal administration of metformin is performed to maintain patients’ adherence in the clinical setting. A recent report revealed that postprandial glucose levels are improved by pre-meal administration of metformin compared with those following post-meal treatment [24]. Our data demonstrated that pre-meal treatment of metformin significantly improved both glucose and TG concentrations compared with post-meal administration (Figure 2C,E). The simultaneous treatment of metformin showed intermediate suppressions both in glucose and TG concentration, which might be due to reduced intraluminal concentration of metformin. The fate of dietary fat involves multiple steps, including gastric emptying, digestion in the intestine, absorption, TG storage in adipose tissue by lipoprotein lipase and fat oxidation in liver and skeletal muscle. In our study, metformin significantly reduced postprandial TG levels by delaying gastric emptying (Figure 3). Regulation of gastric emptying represents the most important brake against nutrient delivery to the intestine, which slows absorption of nutrients in the small intestine. Several studies have reported that delaying gastric emptying reduced postprandial glucose concentrations, while the relationship between delayed gastric emptying and reduced postprandial TG concentrations was unknown [25,26,27]. To the best of our knowledge, this study is the first to demonstrate that the acute effect of metformin on postprandial hypertriglyceridemia is mediated by delayed gastric emptying. The molecular mechanisms underlying this phenomenon are entirely unknown. Further studies are necessary to elucidate the mechanisms and confirm this concept in human subjects.

Three limitations should be noted. First, the dosage of metformin in this study needs to be considered with caution for human applications. We chose 250 mg/kg of metformin based on the glucose-lowering effect. Although delayed gastric emptying might be part of the response to toxicity of this dose, no change in dietary intake or body weight was observed in mice treated with 250 mg/kg of metformin for one week. Furthermore, metformin concentrations in the jejunum can reach levels that are 30–300-fold higher than those in the plasma [28] or other tissues [29]. These facts suggest that clinical dosages in humans might have a similar effect. Second, metformin was administered in two doses (50 or 250 mg/kg/day) with a single administration. The threshold dose for the TG-lowering effect may be determined by graded experiments between 50 and 250 mg/kg. Multiple administrations per day also may impact the TG-lowering effect. Third, we have not directly compared a single administration with an acute effect during chronic administration. The differences in the TG-lowering effect observed in the acute setting may be attenuated, because chronic administration would promote a steady state.

In conclusion, single-dose metformin treatment before olive oil-loading significantly reduced postprandial plasma TG concentrations by delaying gastric emptying. Our results suggest that altered metformin administration timing without additional medication would represent a novel approach for enhancing TG-lowering strategies in patients with T2DM and postprandial hypertriglyceridemia.

## 4. Materials and Methods

### 4.1. Materials

All materials were reagent grade and purchased from Nacalai Tesque (Kyoto, Japan) unless otherwise indicated. Metformin was purchased from Sigma-Aldrich Japan Co. (#D150959, Tokyo, Japan). Glyceryl-1-^13^C tripalmitate was obtained from Tsukishima Foods Industry Co., Ltd. (Tokyo, Japan).

### 4.2. Animals

129S mice were purchased from Jackson Laboratories (Bar Harbor, ME, USA). Male 129S mice aged 8–11 weeks were used for all experiments. The animals were housed under standard conditions with a 12 h light/dark cycle and free access to water and food. The mice were fed a high-fat diet for three weeks (HFD; 60% of total calories from fat, Research diets, Inc. (#D12492, New Brunswick, NJ, USA). All procedures for animal experiments were approved by the Shiga University of Medical Science Animal Care Committee concerning animal experiments (identification code: 2013-5-3, approval date: 20 May 2013) and animals were treated in accordance with the Guidelines of the United States National Institutes of Health [30] regarding the care and use of animals for experimental procedures and the Guidelines of Shiga University of Medical Science for the care and use of laboratory animals.

### 4.3. Pharmacological Intervention

Metformin was dissolved in water. Mice were given an oral gavage of 50 or 250 mg/kg of metformin or vehicle (water).

### 4.4. Body Weight and Food Intake

Mice were housed in pairs, and body weights and food intakes were determined before and after metformin treatment (50 and 250 mg/kg/day) for one week during the light cycle. Food intake (grams per day) was measured daily for seven days. Diet for fed mice contained 60% of total calories from fat.

### 4.5. Oral Glucose Tolerance Test (OGTT)

Glucose (1 g/kg) was administered orally following a 6-h fast. Tail vein bleeds were used to measure plasma glucose levels at 0, 30, 60, 90, and 120 min after glucose-loading.

Experiment 1: Glucose-lowering effect of different metformin dosages administered for one week

OGTT were examined after one week of metformin treatment (50 mg/kg and 250 mg/kg) in 129S mice fed a HFD for three weeks. Final administration of metformin was performed 6 h before glucose-loading.

Experiment 2: Glucose-lowering effect of different timing of single-dose metformin administration

OGTTs were examined at the different administration setting in 129S mice fed a HFD for three weeks. Metformin (250 mg/kg) was administered 1 h before, concurrently with, and 0.5 h after glucose loading.

### 4.6. Oral Lipid Tolerance Test (OLTT)

OLTTs were performed as previously described with a minor modification [31,32,33]. Briefly, olive oil (0.4 mL) was administered orally following a 6-h fast. Tail vein bleeds were used to measure plasma TG at 0, 60, 120, and 180 min after olive oil-loading.

Experiment 1: TG-lowering effect of metformin administration for 1 week

OLTTs were examined after administration of metformin (250 mg/kg) for one week in 129S mice fed a HFD for three weeks. The final metformin administration was performed 6 h or 1 h before oil-loading.

Experiment 2: TG-lowering effect of metformin with different administration periods

To compare the acute and chronic effects of metformin administration on postprandial lipid metabolism, mice were treated with a single or one-week administration of metformin. The final metformin administration (250 mg/kg) was performed 1 h before oil loading.

Experiment 3: TG-lowering effect of a single metformin dose with different administration timings

OLTTs were examined in 129S mice fed a HFD for three weeks. Metformin (250 mg/kg) was administered 1 h before, concurrently with, and 0.5 h after glucose loading.

### 4.7. ApoB-48 Secretion from Primary Small Intestinal Enterocytes

ApoB secretion from primary small intestinal enterocytes was measured as previously described with minor modifications [34]. Briefly, olive oil (0.4 mL) was orally administered 1 h after administration of a single-dose of metformin. The small intestine was extracted and cut into 2 × 2 mm^2^ fragments. Fragment were incubated in ice cold MatriSperse Cell Recovery Solution (Corning, #354253, Corning, NY, USA) at 4 °C for 8 h without agitation. The obtained fragments were transferred to a plastic culture dish containing 7 mL of ice-cold MatriSperse Cell Recovery Solution and incubated at 4 °C for 8 h without agitation. The dish was then gently shaken to separate the villi, and the villi suspension was washed twice in ice-cold PBS (−) (180 g, 5 min). After the final spin, the villi were resuspended in Dulbecco’s Modified Eagle’s medium with 10% fetal bovine serum and 0.2 U/mL insulin in the culture dishes (1 × 10^6^ cells/well). We measured the amount of apoB-48 (Wako Chemicals Co., #K23300R, Takasaki, Japan) secreted from primary cultured enterocytes into the medium for 120 min after resuspension. Immunoblotting using an apoB-48 antibody was performed on the triacylglycerol-rich lipoprotein fractions by SDS-PAGE analysis.

### 4.8. Analysis of the Actual Ratio of ^13^CO_2_ to ^12^CO_2_ in Sampled Gas

Preparation of glyceryl-1-^13^C tripalmitate, sampling of expired gas and analysis of the actual ratio of ^13^CO_2_ to ^12^CO_2_ in sampled gas were performed as previously reported [35]. After three weeks of HFD-feeding, ^13^C-labeled triacyl palmitic acid (0.4 mL) was administered orally following a 6-h fast to 129S mice with 1 h pre-treatment of metformin (250 mg/kg) or no medication. The administered ^13^C-labeled fatty acids were beta oxidized and finally catabolized to ^13^CO_2_ in the citric acid cycle in mitochondria. Therefore, changes in the ^13^C/^12^C ratio (Δ^13^C) in expired CO_2_ from mice indicated the degree of beta oxidation in the whole body.

### 4.9. Statistical Analyses

Results are expressed as mean ± SEM. Student’s *t* test was used for comparisons between two groups. One way ANOVA and a subsequent post hoc Tukey test were used to determine the significance of differences for multiple comparisons. *p-*values < 0.05 were considered statistically significant. All animal experiments were performed with *n* = 3–12 as indicated in the individual figure legends.
